# Aberrant neuronal differentiation is common in glioma but is associated neither with epileptic seizures nor with better survival

**DOI:** 10.1038/s41598-018-33282-5

**Published:** 2018-10-08

**Authors:** Christoph Patrick Beier, Tine Rasmussen, Rikke Hedegaard Dahlrot, Helene Broch Tenstad, Julie Slinning Aarø, Mai Froberg Sørensen, Sólborg Berglind Heimisdóttir, Mia Dahl Sørensen, Per Svenningsen, Markus J. Riemenschneider, Dagmar Beier, Bjarne Winther Kristensen

**Affiliations:** 1 Department of Neurology, Odense University Hospital, Odense, Denmark; 2 Department of Pathology, Odense University Hospital, Odense, Denmark; 3 Department of Clinical Research, University of Southern Denmark, Odense, Denmark; 4 Department of Oncology, Odense University Hospital, Odense, Denmark; 5 Department of Molecular Medicine, University of Southern Denmark, Odense, Denmark; 6 Department for Neuropathology, Regensburg University Hospital, Odense, Germany

## Abstract

The mechanisms of glioma-associated seizures (GAS) have yet to be fully elucidated. Proneural subtype, isocitrate dehydrogenase 1 (IDH1) mutations, and epileptic seizures are closely associated suggesting that aberrant neuronal differentiation contributes to glioma-associated seizures. In a population-based cohort (n = 236), lack of stem cell marker expression (nestin, musashi) was significantly associated with IDH1 mutations and GAS at diagnosis. *In vitro* data suggested an association of IDH1 mutations and a more differentiated phenotype. Out of eight glioma stem cell (GSC) lines, seven revealed positivity for the synaptic marker protein synaptophysin. Three had synapse-like structures identified by electron microscopy and were either vGlut1 (glutamatergic) or GAD67 (GABAergic) positive. *In vivo*, >10% synaptophysin-positive tumour cells were present in >90% of all gliomas. Synaptophysin expression was associated with proneural subtype and vGlut1 expression, suggesting that most synapse-like structures in glioma are glutamatergic. However, we found null associations between vGlut1 protein/mRNA expression and survival, GAS at onset, development of GAS after resection, and refractory GAS. Synapse-like structures were neither functional nor activated by spontaneous action potentials or cellular networks. Thus, aberrant neuronal differentiation including glutamatergic synapse-like structures is detectable in glioma but is associated neither with epileptic seizures nor with better survival.

## Introduction

The treatment and prognosis of patients diagnosed with glioma of all grades has been improved in the last two decades, e.g. due to the introduction of temozolomide as standard of care for glioblastomas (GBM)^1^ and the combination of radiotherapy and chemotherapy with procarbacin/lomustine/vincristine in low-grade glioma^2^. The number of long-term survivors has accordingly increased and the management of neurological symptoms will therefore become increasingly important in these patients.

Epileptic seizures substantially interfere with the quality of life of glioma patients, especially long-term survivors, irrespective of tumour grade, and are strongly associated with neuropsychological deficits^3,4^. Glioma differs from other kinds of brain tumours due to its increased epileptogenicity with up to 80% of patients suffering from epileptic seizures. There is a substantial lack of valuable epidemiological data, but clinical experience and all available data clearly suggest that the risk of developing seizures is substantially higher in patients with glioma as compared to patients with other kinds of non-glial brain tumours, likely due to glioma-specific mechanisms of epileptogenesis^5^. Although several mechanisms have been proposed to explain tumour-associated seizures, the imbalance between excitatory glutamatergic drive and impaired or defect GABAergic inhibition appears to be the crucial mechanism of glioma-associated seizures^6^. On one hand, the balance of glutamate and GABA is affected by reduced chloride potassium symporter KCC2 and increased Na-K-Cl cotransporter NKCC1 expression in the peritumoural tissue, resulting in inverse chloride homeostasis and GABAergic cortical excitation^7^. On the other hand, extracellular glutamate concentrations are increased in glioma patients. Three major mechanisms regulate glutamate secretion in the CNS^8^: vesicular secretion via neuronal synapses, non-vesicular secretion via the Xc-cystine glutamate transporter (SLC7A11, xCT), and glutamate clearance via aminoacid transporters (EAAT1/2). Increased expression of xCT and decreased expression of EAAT1/2 are both associated with increased glutamate concentrations in the cerebrospinal fluid, which is an independent predictor of glioma-associated seizures (GAS)^9^. Substantial additional experimental evidence suggests an important role of xCT in GAS^10,11^. However, xCT expression is only associated with GAS at diagnosis but neither with refractory seizures nor with the development of seizures after resection^12^. This stresses the need for identification of contributing factors causing GAS. So far, it is yet unknown, if vesicular glutamate secretion also contributes to GAS.

Mutations of isocitrate dehydrogenase 1 (IDH1) and rarely of isocitrate dehydrogenase 2 (IDH2), are driver mutations of low-grade glioma and secondary glioblastoma (GBM)^13,14^ and associated with both seizures and better outcome. The molecular mechanism of the strong and reproducible association of IDH1 mutations and epileptic seizures remains unclear^15–17^. Chen *et al*. could recently show that D-2-hydroxyglutarate (D2HG), the product of mutant IDH1, is able to directly activate N-methyl-D-aspartate (NMDA) receptors. Although this provides an elegant explanation for the increased epileptogenicity of IDH1 mutant glioma, the D2HG concentrations needed for activation of NMDA receptors *in vitro* (10 mM) have been higher than the actual D2HG concentrations measured in the CNS of patients (1–6 mM)^16,18^.

Mutations of IDH1 are associated with better outcome, substantially increased risk of developing epileptic seizures, and cause a CPG-island hypermethylation phenotype that is associated with the proneural subtype of glioma^19,20^. The association of outcome, epileptic seizures, IDH1 mutations and the proneural phenotype suggests a pathophysiological link between the factors, especially between proneural differentiation and epileptic seizures. Further evidence supporting the hypothesis that neural differentiation causes seizures came from a study investigating the association of the synaptic protein SV2 and the response of GAS to anti-epileptic treatment with levetiracetam (that specifically binds to SV2A). Unexpectedly, the expression of the synaptic protein SV2A in bulk tumour cells showed an even stronger association with response to levetiracetam than SV2A expression in the infiltration zone, suggesting that neuronal structures within the tumour contribute to epileptogenesis.

In patients, the expression of neuronal markers in glioma remained without prognostic significance in two retrospective cohort studies^21,22]^. Classical serum-cultivated glioma cell lines did not express neuronal markers, and neuronal differentiation *in vitro* was not yet studied in detail. Therefore, we used glioma stem cells (GSC) maintaining the ability to differentiate into cells expressing markers of all three neural lineages^23–25^ to study neuronal differentiation of glioma cells and its association with IDH1 mutations, outcome, and epileptic seizures.

## Results

### Nestin is not an independent prognostic factor for epileptic seizures but associated with IDH1 R132 mutations.

**Figure 1:**
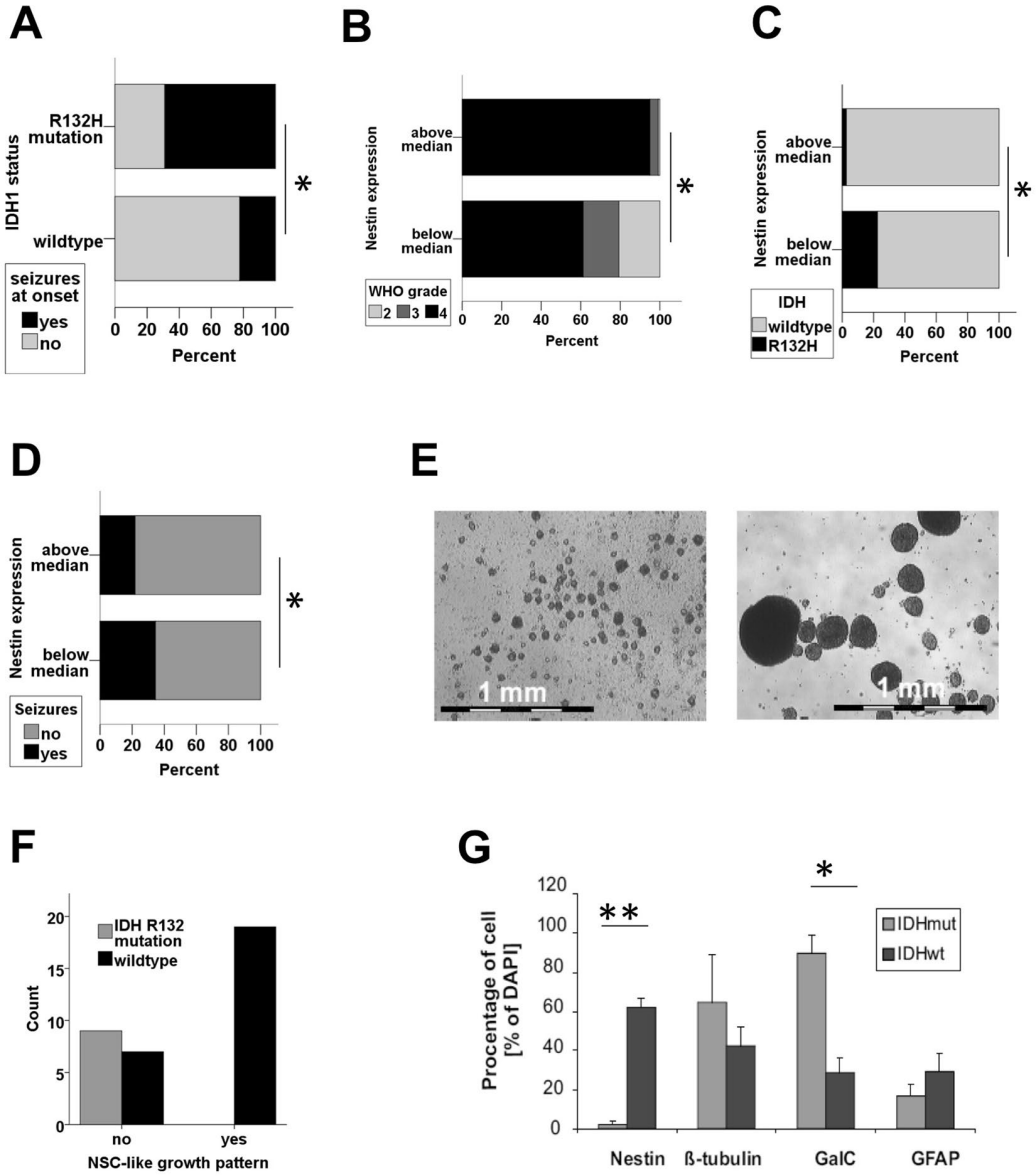
IDH1 wildtype status positively correlates with tumour stem cell properties. (**A**) The risk of seizures at onset is associated with IDH1 R132H mutations (*p < 0.05, chi^2^-test). (**B**,**C**) Distribution of WHO grade (**B**) and IDH mutation status (**C**) in glioma with high and low nestin expression (above and below median, *p < 0.05, chi^2^-test). (**D**) Seizures at onset in tumours with nestin expression above and below median (*p < 0.05, chi^2^-test). (**E**,**F**) Representative images illustrating lineage-restricted progenitor cell-like (to the left, R47) and neurosphere-like growth (to the right, R28, 25-fold magnification each). *IDH* mutation status correlates with the ability to form neurospheres. (***p < 0.0002, chi^2^-test). (**G**) The differentiation pattern of first-passage tumour cell cultures of three gliomas with IDH R132H (R26, R24, R42) mutations and four IDH wildtype gliomas (R11, R18, R22, R28) are given (**p = 0.0004, *p = 0.005, two-sided student’s t-test).

We studied the association of the stem cell markers nestin and musashi-1 with epileptic seizures at diagnosis in a cohort comprising 239 patients with gliomas (WHO II-IV, overview over patients: Supplementary Table 1, previously reported in^26^). IDH R132H mutation was strongly associated with seizures at onset (Fig. 1A). High nestin and high musashi-1 expression were associated with WHO grade (Fig. 1B, Supplementary Fig. 1A) and IDH^wildtype^ (Fig. 1C, Supplementary Fig. 1B). IDH R132H mutation and low WHO grade showed a strong and significant association with epileptic seizures at diagnosis (Supplementary Table 2). In contrast, nestin and musashi-1 expression were associated inversely with epileptic seizures (Fig. 1D, Supplementary Fig. 1C, Supplementary Table 2). Due to the assumed interaction of IDH1 mutation, WHO grade and nestin expression, we performed a multi-variate analysis to identify crucial contributors among the three factors associated with epileptic seizures. Logistic regression showed that only IDH1 R132 mutations but neither nestin expression nor WHO grade remained independently associated with seizures at diagnosis (Supplementary Table 3).

### IDH1 mutations in glioma are associated with a more differentiated phenotype *in vitro*

From a previously studied cohort of >70 glioma patients^23,27,28^, data on IDH status was available for 42 patients (Supplementary Table 4). IDH1 mutated tumours did not show persistent growth of GSC *in vitro* (Fig. 1E,F) and had significantly lower nestin expression (Fig. 1G). The spontaneous *in vitro* differentiation pattern (passage 1, direct after resection) was available from 7 tumours. First passage glioma cells from tumours with IDH1 R132H mutations showed a more restricted differentiation pattern as compared to wildtype gliomas and almost exclusively expressed markers of oligodendrocytes and neurons (Fig. 1G).

### Expression of markers indicating neuronal differentiation in GSC lines

**Figure 2:**
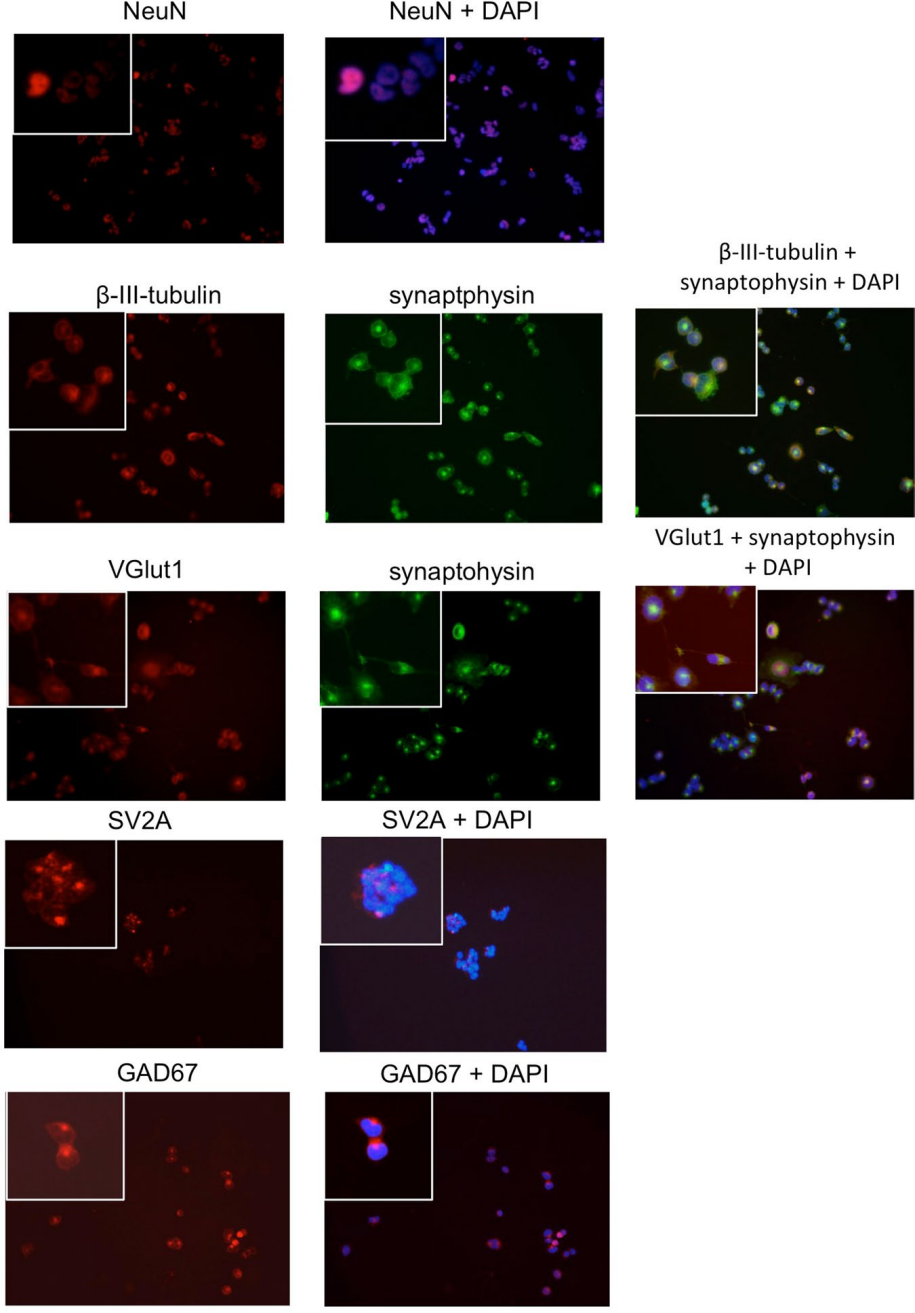
Expression of markers of neuronal differentiation. The entire panel of GSC lines was stained for SV2A, NeuN, vGlut1, GAD67 and synaptophysin. Representative staining of each marker from the GSC line R28 are shown. Single cells are shown with higher magnification in the inset given in the left upper corner. DAPI counterstaining was used to visualize nuclei.

We hypothesized that the more differentiated phenotype of IDH1^R132H^ glioma may contribute to their higher epileptogenicity. We therefore studied the spontaneous aberrant neuronal marker expression in seven primary GSC lines from two different laboratories (CPB and BWK)^23–25^. All GSC lines were cultured using serum-free medium supplemented with EGF and FGF. Cells from all GSC lines expressed markers of neuronal (NeuN, beta-3-tubulin) differentiation to a certain degree. In three GSC lines (R11, R28, T111) a subset of cells showed a strong and consistent neuronal marker expression pattern. They expressed markers for immature neurons (beta-3-tubulin), markers for mature neurons (NeuN) and markers indicative for synapses (synaptophysin, SV2A, Fig. 2, Supplementary Fig. 2, Table 1). The expression pattern was inconsistent in four GSC lines (Table 1).

**Table 1:** Markers of neuronal differentiation in a panel of GSC lines.

	Beta-tubulin	Synapto-physin	SV2A	vGlut1	NeuN	GAD67	Synapse-like structure EM
R8	+	(+)	++	−	−	++	(−)
R11	+++	+++	+++	+	−	(+)	+
R18	+	(+)	+	−	−	+++	(+)
R28	++	+++	+	++	+	+	++
T78	+	(+)	n.a.	−	(+)	++	−
T87	−	+	n.a.	−	−	(+)	(+)
T111	+	+	+++	−	++	++	(+)
Murine NSC	+		n.a.	++	n.a.	++	++

Legend: Immunocytochemistry: −No staining.

(+) few cells/low staining intensity + clear positivity ++ - +++ strong/very strong staining intensity.

Electronmicroscopy: −No structures reminiscent to synapses. (+) vesicles + synapses ++ many synapses.

### Synapse-like structure within GSC lines *in vitro*

**Figure 3:**
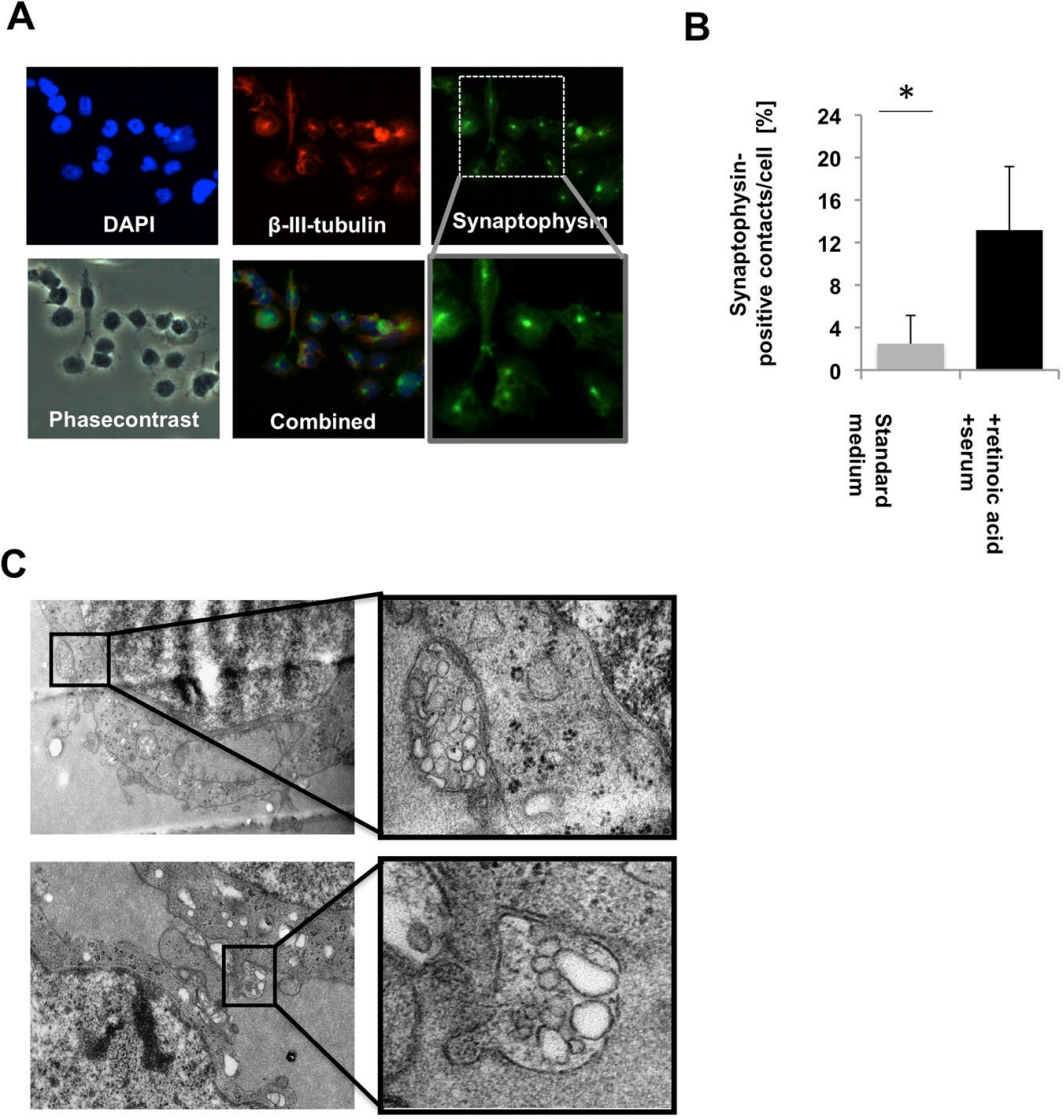
Synapse-like structures within GSC lines. (**A**) DAPI, synaptophysin (high and low magnification), phasecontrast and β-III tubulin staining of GSC line R28 is shown. (**B**) Number of synaptophysin-positive cell-cell contacts under standard culture conditions (serum free medium supplemented with EGF/FGF/B27) and under conditions favoring neuronal differentiation (no growth factors, 1% serum, 0.1% retinoic acid, p < 0.005, student’s-t-test). (**C**) Electronmicroscopy of GSC spheres cultured under standard conditions. Synapse-like structures are shown in higher magnification on the right side.

We found cell-cell contacts with strong positivity for the pan-synaptic markerprotein synaptophysin suggesting synapse-like structures (Fig. 3A) in two GSC lines (R11 and R28 but not in the GSC line T111). Culture conditions known to foster neuronal differentiation in neuronal stem cells (growth factor deprivation, 1% serum, 0.1% retinoic acid), significantly increased the number of synaptophysin positive cell-cell contacts (Fig. 3B). To verify that these synaptophysin-positive cell-cell contacts correspond to synapse-like structures, we used electronmicroscopy of GSC cultured as spheres under standard conditions. Culturing of GSC lines as spheres allows the analysis of the direct interaction of glioma cells. The three GSC lines with substantial neuronal differentiation but none of the other GSC lines showed structures resembling synaptic vesicles. The GSC lines R11 and R28 that had spontaneous synaptophysin-positive cell-cell contacts also showed microstructures reminiscent to synapses (Fig. 3C, Table 1).

### Synapse-like structures within GSC lines *in vivo* and in human glioma

**Figure 4:**
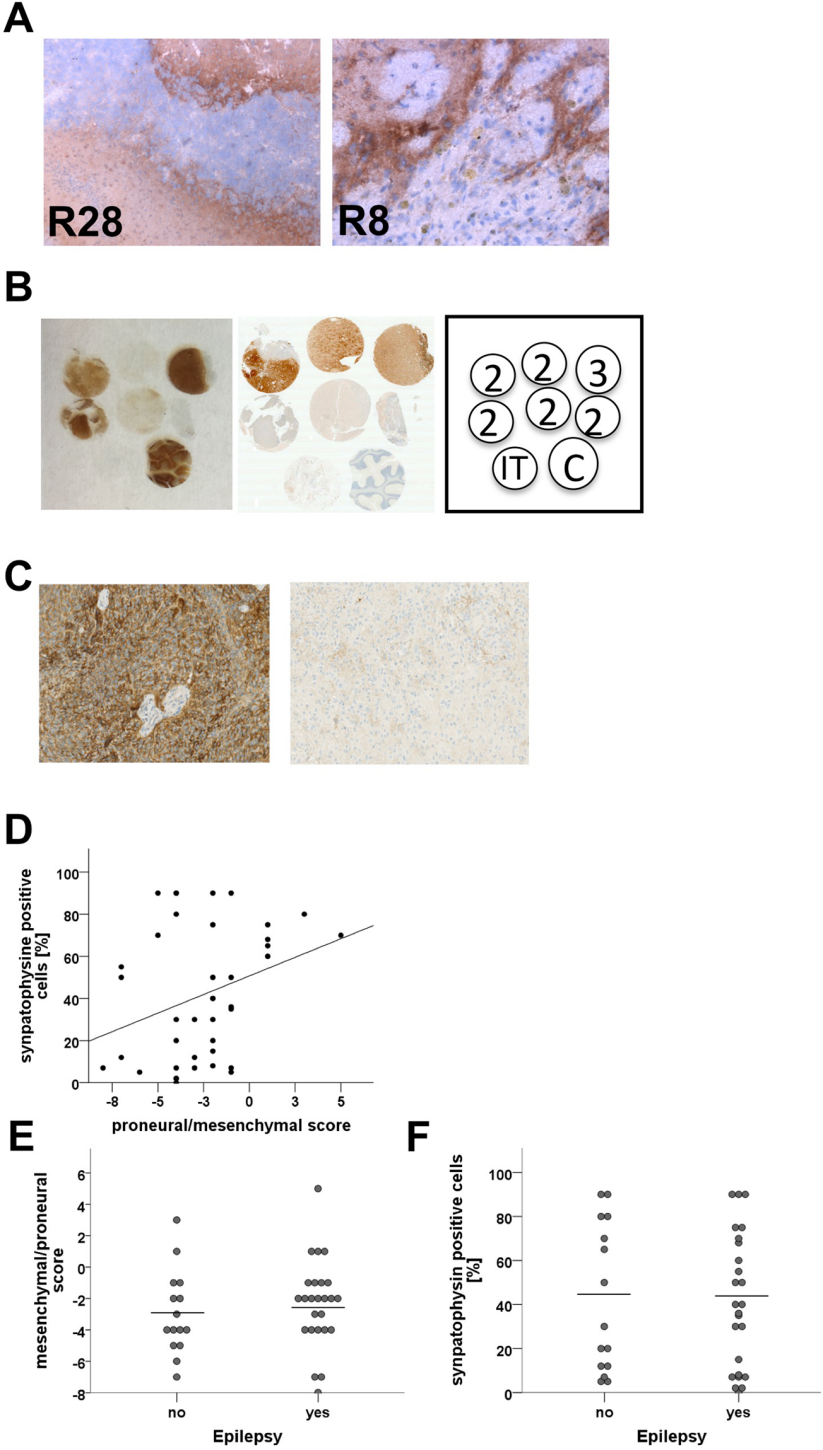
Synapse-like structures *in vivo*. (**A**) Immunostaining detecting human synaptophysin in xenograft tumours (R8, R28). (**B**) Synaptophysin immunostaining (left side) and IDH1 immunostaining (right side) of a tissue *microarray* containing WHO grade 2 tumours (2) and WHO grade 3 tumour (3), cerebellum as positive control (C), and intestine as negative control (IT) for the synaptophysin staining. (**C**) Representative synaptophysin immunostaining of human IDH wildtype glioblastoma samples with high (left) and low synaptophysin expression (right). (**D**) Correlation of synaptophysin expression with markers indicative for proneural (proneural-mesenchymal score >0) and the mesenchymal subtype (proneural-mesenchymal score <0, Pearson r = 0.3, p = 0.05). (**E**) Association of epileptic seizures and markers indicative for proneural (proneural-mesenchymal score >0) and the mesenchymal subtype (proneural-mesenchymal score <0 (p = 0.3, Mann-Whitney U-test). (**F**) Association of synaptophysin expression with epileptic seizures (p = 0.9, Mann-Whitney U-test).

To exclude that the synapse-like structures correspond to a cell culture artefact, we further analyzed tumour material from xenograft tumours from the GSC lines R8 (slightly synaptophysin positive) and the GSC line R28 (strongly synaptophysin positive). Both xenograft tumours showed substantial positivity for human synaptophysin mainly in the invasion zone and in the boundaries of the tumours (Fig. 4A). To further confirm the presence of synaptophysin-positive glioma cells *in vivo*, we used a tissue-microarray including three IDH1 positive gliomas. Three out of six tumours were strongly positive for synaptophysin, three were negative (Fig. 4B).

Synaptophysin expression was also common in IDH^wildtype^ glioblastoma. In a cohort of 40 samples, 29 out of 40 IDH^wildtype^ tumours expressed synaptophysin (Fig. 4C). Immunostaining with histological markers indicative for the proneural and mesenchymal subtype (described in^29^) showed, a significant association with the proneural subtype but synaptophysin was also detectable in mesenchymal GBM (Fig. 4D). There was no association of epileptic seizures with proneural phenotype or synaptophysin expression (Fig. 4E,F).

### Synapse-like structures within GSC lines may be glutamatergic or GABAergic

**Figure 5:**
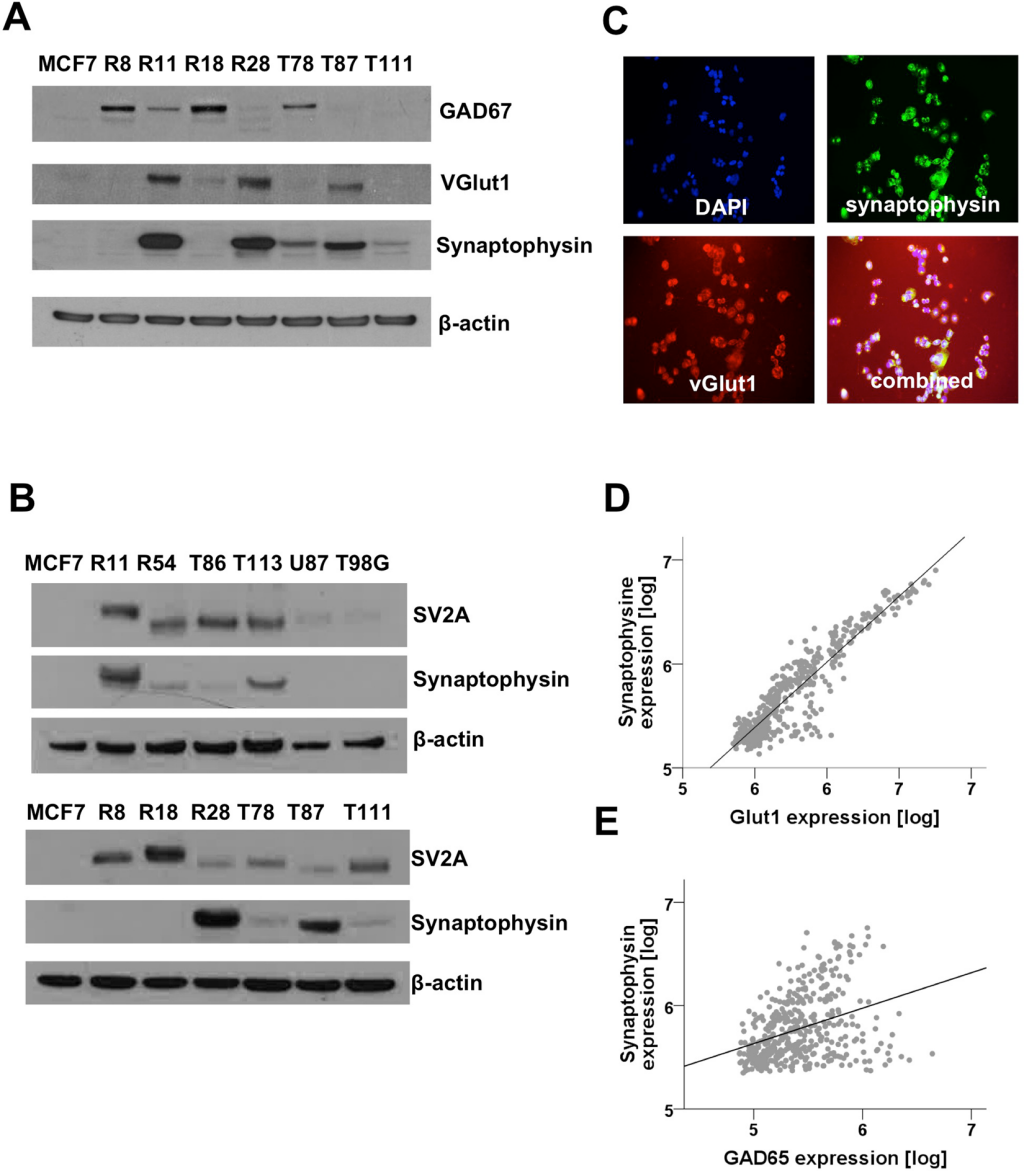
Glutamatergic and GABAergic glioma cells. (**A**,**B**) Westernblot of GSC lines and control samples (breast cancer cell line MCF7, classical glioma cell lines T98G, U87). Blots were probed with antibodies against GAD67, synaptophysin, SV2A and vGlut1. Actin was used as loading control. (**C**) Immunocytochemistry against vGlut1 and synaptophysin. DAPI was used to label the nuclei. (**D**,**E**) Correlation of synaptophysin expression with vGlut1 (Pearson r = 0.92, p < 0.001) and GAD67 (Pearson r = 0.36, p = 0.001) using mRNA expression data from the RemBranDT database (accessed: 09 January 2018).

Glutamate (excitatory) and GABA (inhibitory) are the two most important neurotransmitters in the human brain. To detect glutamatergic synapses, we used the well-establish marker protein vGlut1 (SLC17A7), which is a vesicular protein that transports glutamate into the synaptic vesicle. In our panel of seven GSC lines, three expressed vGlut1. Another four were positive for GAD67, the crucial protein in the biosynthesis of GABA and an established marker for GABAergic neurons (Fig. 5A). One of the GAD67 positive GSC lines was also positive for vGlut1, however, none of the GAD67-positive GSC lines studied had detectable synapse-like structures when studied using electron microscopy (Table 1). Notably, all GSC lines expressed the synaptic protein SV2A, which binds to levetiracetam, to some extent. SV2A was expressed in both glutamatergic and GABAergic synapses and varied substantially between the GSC lines. The classical, serum cultured glioma cells lines like T98G or U87 as well as the breast cancer cell lines MCF7 did not express SV2A (Fig. 5B). *In vitro*, the vesicular transporter vGlut1 co-localized with synaptophysin in both double-positive GSC lines (Fig. 5C). *In vivo*, vGlut1 mRNA expression correlated very strongly with synaptophysin expression in *microarrays* available to the RemBraNDT (REpository for Molecular BRAin Neoplasia DaTa) database (Pearson r = 0.92, p < 0.001, Fig. 5D). In contrast, expression of GAD67 mRNA was less associated with synaptophysin mRNA expression (Pearson r = 0.36, p < 0.001, Fig. 5E). Analysis of the TCGA database available at the same repository gave similar results (Synaptophysin/vGlut1: Pearson r = 0.85, synaptphysin/GAD67: Pearson r = 0.38).

### Lack of association of vGlut1 expression and epileptic seizures

**Figure 6:**
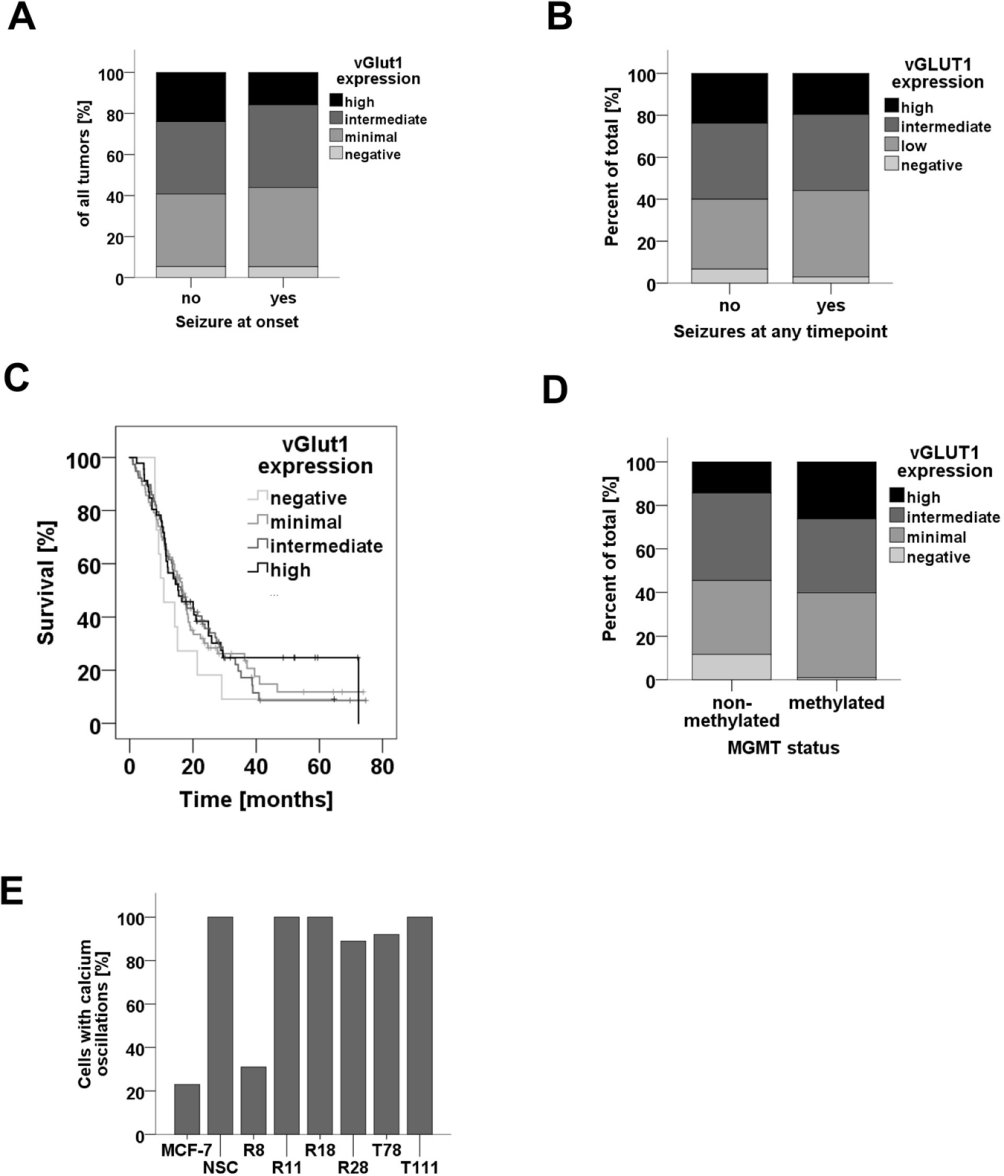
Association of vGlut1 expression with clinical characteristics. (**A**,**B**) Risk of seizures at onset (**A**) and at any time point during disease (**B**) in patients with high, intermediate, low and no vGlut1 expression. (**C**) Survival of patients with high, intermediate, low and no vGlut1 expression (p = 7., Log-Rank test). (**D**) MGMT methylation status in patients with high, intermediate, low and no vGlut1 expression. (**E**) Proportion of cells with spontaneous changing intracellular calcium concentrations within 5 min periods.

Given that glutamate is the strongest and most common excitatory neurotransmitter in the human brain, we hypothesized that neuronal differentiation with a “glutamateric phenotype” may contribute to GAS. We therefore studied a large, representative cohort of 224 patients for vGlut1 expression and seizures at onset (Patients characteristics: Supplementary Table 5). However, the expression of vGlut1 was neither associated with IDH1 status nor with tumour grade (data not shown). Further, we found no association with seizures at onset (Fig. 6A), development of seizures at any time point (Fig. 6B), or survival (Fig. 6C). vGlut1 expression was associated with MGMT methylation pattern (Fig. 6D).

### GSC lines with marked neuronal differentiation are not excitable and do not show spontaneous action potentials

To further understand why these vesicular structures filled with glutamate did not influence the odds for developing epileptic seizures, we studied if glioma cells with neuronal differentiation are able to release glutamate. In mature neurons, synaptic release of glutamate is triggered by calcium influx due to voltage-dependent calcium channels. We therefore studied intracellular calcium levels using Fluo4. Time-lapse imaging in a cell culture incubator with stable temperature and oxygen concentrations revealed that five out of six GSC lines studied showed spontaneous calcium changes in intracellular calcium concentrations that were not seen in the breast cancer cell lines MCF7 (Fig. 6E, Supplementary Film 1). The membrane potentials in single R28 cells were depolarized (−28 ± 5 mV, n = 4) and stable, indicating that the changes in intracellular calcium were not due to spontaneous action potentials (data not shown). Moreover, the changes in intracellular calcium were not consistently inhibited or induced by high potassium concentration (data not shown) and the analysis of close adjacent cells clearly indicated that glioma cells did neither create cellular networks nor consistently modified calcium levels in neighbouring cells (Supplementary Film 2).

## Discussion

Aberrant neuronal marker expression - alone or in combination with glial markers - is well established in GSC lines and was also observed *in vivo*^21,23,24,30^. We here provide evidence that a subset of glioma cells display more advanced neuronal differentiation *in vitro* and *in vivo* that went beyond aberrant marker expression but included synapse-like structures with the ability to generate non-functional synapse-like direct cell-cell contacts among glioma cells.

Our data did not substantiate the hypothesis that the (pro-)neural differentiation was crucial for or at least contributed to GAS. We challenged the hypothesis using several approaches (e.g. association of proneural phenotype and seizures in IDH^wildtype^ GBM), correlation of synaptophysin and seizures in IDH^wildtype^ GBM). None of the results supported the hypothesis. We therefore only focused on gliomas with a glutamatergic differentiation but did not find any association with seizures either. Given that the proneural phenotype is associated with the polymethylator-phenotype^19^, the identified association of neuronal differentiation, glutamatergic synapse-like structures, and MGMT methylation was expected. In summary, the provided data falsified the working hypothesis. For obvious reasons, it is impossible to exclude a yet unknown mechanism associated with neuronal differentiation and contributing to GAS in minor subgroups within the different cohorts studied. However, because we did not find consistent or suggestive trends in our data in support of the working hypothesis, we do not assume having overlooked a biological relevant effect.

Our data suggests that proneural differentiation and GAS are both secondary to IDH1 R132H gain-of-function mutations. The most plausible explanation for GAS in IDH^R132H^ glioma is directed stimulation of NMDA receptors by D2HG^16^. We assume that neuronal differentiation is likely secondary to the epigenetic changes resulting from D2HG induced hypermethylation as suggested by data from Noushmehr *et al*.^19^. In line with other studies, we did not find a prognostic relevance of neuronal differentiation as indicated by vGlut1 expression^21,22^.

Although our data consistently falsify the working hypothesis, the amount and degree of neuronal differentiation within glioma was surprising. Here we describe and characterize for the first time synapse-like structures within glioma cells *in vitro* and *in vivo*. The GSC lines with consistent neuronal differentiation used for our experiments have been derived from typical primary GBM and were able to form GBM-like lesions after transplantation into nude mice^23,29,31^. By choosing GSC lines from two laboratories, we reduced the risk of contamination or other technical artefacts. The biological significance of this neuronal differentiation remains unknown and the results of several experiments were unanticipated (e.g. expression of synaptophysin in the invasion zone of xenotransplants only). It may represent an epiphenomenon that indicates that the cells of origin of GBM as well as the stem cell-like tumour cells (“cancer stem cells”) driving tumour growth are pluripotent with a variable degree of neuronal differentiation. This would be in line with a recent study using a murine glioma model describing that all glioma derive from proneural progenitors^32^. From a broader perspective, it is likely that we characterized the cellular correlate to the proneural/neural glioma subtype identified in the context of several large-scale attempts to subclassify glioma^19,20,28,33–35^. As in our study, the translational significance of the different subtypes identified in the last decade remains vague despite evidence suggesting some importance for treatment response^36–39^. Although we did not assess the molecular status of the glioma samples used in detail, it appears likely that the assumed higher incidence of seizures in proneural glioma is secondary to IDH mutations and therefore adds GAS to the list of clinical features not associated with the molecular well-defined subtypes.

In summary, our study provides evidence suggestive for the existence of an advanced neuronal differentiation of glioma cells and showed that neuronal differentiation of glioma cells is associated neither with survival nor with seizures at onset.

## Material and Methods

### Patient cohorts

Cohort Fig. 1 and Supplementary Fig. 1: This retrospective study examined all adult patients with newly diagnosed glioma in the Region of Southern Denmark between 2005 and 2009 (n = 433). For 239 of these patients, sufficient tumour tissue was available. The histological diagnosis was made using the 2007 WHO criteria^40^. Three patients with WHO grade 1 glioma were excluded. Data on seizures at onset were available for all patients. The patient cohort and nestin staining has been published previously^26^ (Ethic Committee approval: S2DO90o80, Danish Data Protection Agency: 2009-41-3070). All experiments in this paper with patient material/data were performed in accordance with relevant national and local guidelines and regulations.

Cohort Fig. 4D: This retrospective study examined patients diagnosed with adult IDH^wildtype^ GBM at Odense University Hospital between January 2009 and December 2012 (n = 40). The GBM were chosen consecutively by starting at the last patient diagnosed in 2012 and going backwards until we had a total of 40 patients (Ethic committee approval: S-20110022, Danish Data Protection Agency: 16/32269).

Cohort Fig. 6: The cohort includes all consecutive glioma samples resected in the Department of Neurosurgery, Odense University Hospital, with sufficient high quality material for tissue microarrays. The histological diagnosis was made using the 2007 WHO criteria^40^. Demographic and survival data, data on seizures at onset, development of seizures at any time point, data on molecular markers (methyl-guanidin-methyl-transferase (MGMT) promoter, IDH1 status) were systematically assessed based on the patients records. In the same cohort, expression of xCT was determined, which was published elsewhere^41^ (Ethic committee approval:. S-20150148, Danish Data Protection Agency: 16/32269).

### Generation of GSC lines

Between 2005 and 2010, >70 GSC lines have been established and characterized using tumour samples from glioma WHO grade 2-4.^23,27,28^. This was approved by the local ethics committee (no: 05/105, University of Regensburg, Germany). The preparation of GSC lines was previously described in^23,25^. In brief, fresh tumour material was dissociated into a single cell suspension and the cells were seeded in serum-free medium supplemented with 20 ng/ml EGF and FGF in addition to LIF and B27 supplement. GSC lines were cultured at least 4 weeks and the growth pattern was analyzed at least once a week. The growth pattern described refers to the initial growth pattern of GSC lines during the first 5 passages. Generation of GSC lines T111 and T78 were previously described^42^. For all other experiments, GSC lines were cultured in serum-free medium supplemented with 20 ng/ml EGF, 20 ng/ml FGF, and 1% B27 supplement.

### Histology

The IDH1 status of the GSC lines was determined by sequencing as described in^43^. In all other experiments, the IDH R132H mutation was detected as previously described^44^ using the mIDH1R132H antibody (mIDH1R132H, clone H14, Dionova, 1:100).

Nestin and musashi-1 positive tumour cells were identified as previously described (nestin 196908 antibody, R&D systems (1:100) and musashi-1 clone 14H1, MBL International, 1:200)^26,45^. Slides were stained on the AutostainerPlus platform (Dako, Glostrup, Denmark) and DyLight 650 (1 + 100 Fisher Scientific) was used for detection of nestin. The fraction of nestin positive tumour cells was identified using quantitative image analysis with continuous estimates of staining in the Visiomorph module of the Visiopharm Integrator System (Visiopharm, Hørsholm, Denmark).

Three-micrometer sections of paraffin-embedded GBM samples were stained using synaptophysin antibody from NovoCastra (NCL-SYNAP-299, clone 27G12, 1:50) on an automated immunostainer (BenchMark Ultra IHC/ISH staining system, Ventana Medical Systems, USA). OLIG2, NeuN, DLL3, YKL40, CD44, and VEGF were stained as described in detail in^46^. All samples were scored blinded (MFS, SBH). Synaptophysin was evaluated with respect to antigen expression alone, and scored from 0–100 according to percent positivity. OLIG2, NeuN, DLL3, YKL40, CD44, and VEGF was scored as described in^29^. In brief, 5 positions per slide were scored from each marker (0: no expression; 1: <10% expression; 2: 10%–49% expression, 3: 50%–90%, 4: >90%). The sum of the means of the mesenchymal markers was subtracted from the sum of the means of the proneural markers.

### *In vivo* tumour model

The tumour model was described previously, in brief 50.000-100.000 tumours cells were suspended in 2 µl saline and injected intracranially injected into T-lymphocyte-deficient NMRI mice as described in^23,31^. The procederes were conducted in accordance with German law governing animal care and approved by the local authorities (Az. 621-2531.1-04/03, Regierung der Oberpfalz, Germany). 10 µm slides were stained against synaptophysin (NovoCastra (NCL-SYNAP-299, clone 27G12, 1:100) and counter stained with H&E using standard protocols.

### Immunocytochemistry

Immunocytochemistry of glioma cells plated on cover slips coated with poly-ornithine and laminin was performed as described in^28^. The following antibodies were used in a dilution of 1:100 if not otherwise specified: beta-3-tubulin: Promega G7121 (1:1000), GFAP: DAKO Z 0334, Nestin: Chemicon MAB5326 (1:500), NeuN: Chemicon MAD377 (1:50), SVA2: Abcam ab49572 (1:100), GAD67: ThermoFisher PA1-37733 (1:100), vGlut1: Abcam ab193595 (1:100), synaptophysin: Novacastra NCL-SYNAP-299 (1:50).

### Electronmicroscopy

Electronmicroscopy was performed according to standard procedures in the Department of Pathology, Odense University Hospital. In brief, cells were centrifuged, washed using 0.1 phosphatbuffer and fixed using 2% glutaraldehyd. After fixation, pellets were stained using 1% osmiumtetraoxid and embedded in epoxy and analyzed using JEOL JEM-1400 electronmicroscope.

### Westernblot

Westernblots were performed according standard procedures. The following antibodies were used for probing: beta-actin: Sigma A5441 (1:1000), Promega G7121, SVA2: Abcam ab49572 (1:1000), GAD67: ThermoFisher PA1-37733 (1:1000), vGlut1: Abcam ab193595 (1:1000), synaptophysin: Novacastra NCL-SYNAP-299 (1:1000).

### Current Clamp

Membrane potentials in GSC cells were recorded using the current-clamp mode of the Axopatch-amplifier (Axon Instruments, Foster City, CA, USA) and the Clampex 9.2 data acquisition software (Axon Instruments). Membrane potentials was measured after obtaining the whole-cell configuration and measured for minutes to test for spontaneous changes. The solution in the pipette was (in mmol/l): 135 K-glutamate; 10 KCl; 10 NaCl; 1 MgCl2; 10 HEPES; 0.3 NaGTP; 0.5 MgATP and 2 EGTA. Bath solution was (in mmol/l): 10 HEPES-NaOH, 140 NaCl, 2.8, KCl, 1 MgCl2, 2 CaCl2, 11 glucose, and 10 sucrose. Experiments were performed at room temperature.

### Microarray analysis

We used the publically available Repository for Molecular BRAin Neoplasia DaTa (REMBRANDT, http://www.betastasis.com/glioma/rembrandt/) that is based on 524 Affymatrix U133 2.0 plus microarrays to investigate the correlation of vGlut1 and synaptophysin expression. The databased was accessed 09 January 2018 and data was analysed using SPSS 24.0.

### Statistics

All statistical analyses were performed used SPSS 24.0. The statistical test used is given in the legend to the respective Figures.

## Electronic supplementary material

Supplementary data
