# Supplementary material for: Aberrant neuronal differentiation is common in glioma but is associated neither with epileptic seizures nor with better survival
Source: Sci Rep. 2018 Oct 8;8:14965. doi: 10.1038/s41598-018-33282-5 (PMC6175915; doi:10.1038/s41598-018-33282-5)
Supplement: Supplementary file 1 — Supplementary data [file 41598_2018_33282_MOESM1_ESM.pdf]

Supplementary Information

"Aberrant neuronal differentiation is common in glioma but is associated neither with epileptic seizures nor with better survival"

by

Christoph P. Beier[1,3], Tine Rasmussen[1,2], Rikke Hedegaard Dahlrot[4], Helene Broch Tenstad[1,2], Julie Slinning Aarø[1,2], Mai Froberg Sørensen[1,2], Sólborg Berglind Heimisdóttir[1,2], Mia Dahl Sørensen[2,3], Per Svenningsen[5], Markus J. Riemenschneider[6], Dagmar Beier[1,3], Bjarne Winther Kristensen[2,3]

[1] Department of Neurology, Odense University Hospital, Denmark

[2] Department of Pathology, Odense University Hospital, Denmark

[3] Department of Clinical Research, University of Southern Denmark, Odense, Denmark

[4] Department of Oncology, Odense University Hospital, Denmark

[5] Department of Molecular Medicine, University of Southern Denmark

[6] Department for Neuropathology, Regensburg University Hospital, Germany

Supplementary Table 1: Patient characteristics

	N (% of total)
**Age at diagnosis (years)**	64 (19-82)
**Average survival (months)**	12 (0.03-139)
**Overall survival (2 years)**	66 (27%)
**Overall survival (5 years)**	26 (11%)
**Gender**	
- **Male**	142 (59%)
- **Female**	97 (41%)
**IDH1 R132H status**	
- **Wildtype**	206 (87.7%)
- **Mutated**	29 (12.3%)
**Histological diagnosis (WHO)**	
- **Low-grade glioma (II)**	25 (10.6%)
- **Anaplastic glioma (III)**	26 (11.1%)
- **Glioblastoma (IV)**	184 (78.3%)
**MGMT methylation**	
- **yes**	79 (33%)
- **no**	76 (32%)
- **unknown**	84 (35%)
**Seizures of onset**	
- **yes**	66 (28.1%)
- **no**	169 (71.9%)

Supplementary Table 2: Analysis patients with and without documented epileptic seizures at onset

	Epileptic seizures	No epileptic seizures	Odds ratio	p-value
**Nestin expression**				
Above average	26	93	1.8	0.03[1]
Below average	40	76	(CI: 1.1-3.3)	
**IDH 1 status**				
Wildtype	46	160	7.7	
R132H mutation	20	9	(CI: 3.2-18.1)	<0.001[1]
**WHO grade**				
I	0	3	n.a.	<0.001[1]
II	18	7		
III	8	18		
IV	40	144		

[1] Chi-squared test

Supplementary Table 3: Multivariate analysis (logistic regression analysis) of putative factors associated with seizures at diagnosis

	Odds ratio	95% Confidence interval	p-value
**Nestin expression**			
**High vs. low**	0.9	0.5-1,8	0.85
**Tumor grade**			
**Grade II**	4.8	0.4-63.3	0.22
**Grade III**	1.2	0.09-14.9	0.91
**Grade IV**	1.4	0.1-16	0.81
**IDH1**			
**Wildtype vs. R132H mut.**	4.8	1.6 – 14.4	0.005

Supplementary table 4: Synopsis of histological diagnosis, *IDH* mutation status, growth pattern and CD133 expression

tumor ID	histological diagnosis	WHO grade	IDH1 mutation status (Codon 131-132)	IDH2 mutation status (Codon 172)	Growth pattern
R3	primary GBM	IV	(wt)	n.a.	Neurosphere-like
R7	primary GBM	IV	n.a.	Wt	Neurosphere-like
R11	primary GBM	IV	Wt	Wt	Neurosphere-like
R17	primary GBM	IV	Wt	Wt	Neurosphere-like
R18	primary GBM	IV	Wt	Wt	Neurosphere-like
R22	primary GBM	IV	n.a.	Wt	Neurosphere-like
R28	primary GBM	IV	Wt	Wt	Neurosphere-like
R30	primary GBM	IV	Wt	Wt	Neurosphere-like
R34	primary GBM	IV	Wt	n.a.	Neurosphere-like
R35	primary GBM	IV	Wt	Wt	Neurosphere-like
R36	primary GBM	IV	Wt	Wt	Neurosphere-like
R40	primary GBM	IV	Wt	Wt	Neurosphere-like
R44	primary GBM	IV	Wt	Wt	Neurosphere-like
R58	primary GBM	IV	Wt	Wt	Neurosphere-like
R54	primary GBM	IV	Wt	Wt	Neurosphere-like
R52	primary GBM (oligo component)	IV	Wt	Wt	Neurosphere-like
R6	primary GBM	IV	n.a.	Wt	no growth
R29	primary GBM	IV	Wt	Wt	no growth
R38	primary GBM	IV	Wt	Wt	no growth
R50	primary GBM	IV	Wt	Wt	no growth
R55	primary GBM	IV	Wt	Wt	no growth
R60	primary GBM	IV	Wt	Wt	no growth
R61	primary GBM	IV	Wt	Wt	no growth
R8	primary GBM	IV	Wt	Wt	Neurosphere-like (Adherent)
R43	primary GBM	IV	Wt	Wt	Neurosphere-like (Adherent)
R46	primary GBM	IV	Wt	Wt	Neurosphere-like (Adherent)

R49	primary GBM	IV	Wt	Wt	Neurosphere-like (Adherent)
R53	Primary GBM	IV	Wt	Wt	Neurosphere-like (Adherent)
R5	primary GBM	IV	Wt	Wt	n.a.
R13	primary GBM	IV	Wt	Wt	n.a.
R14	primary GBM	IV	Wt	n.a.	n.a.
R25	primary GBM	IV	Wt	Wt	n.a.
R10	secondary GBM	IV	mut	Wt	no growth
R41	secondary GBM	IV	mut	Wt	no growth
R45	secondary GBM	IV	mut	Wt	no growth
R51	secondary GBM	IV	mut	Wt	no growth
R33	anaplastic oligoastrocytoma	III	Wt	Wt	Neurosphere-like, partially also progenitor cell-like
R23	anaplastic astrocytoma	III	Wt	Wt	no growth
R57	anaplastic oligoastrocytoma	III	n.a.	Wt	no growth
R47	anaplastic astrocytoma	III	mut	(wt)	Progenitor cell-like
R24	anaplastic oligoastrocytoma	III	mut	(wt)	Progenitor cell-like
R31	anaplastic oligoastrocytoma	III	mut	Wt	Progenitor cell-like
R42	anaplastic oligodendroglioma	III	mut	Wt	Progenitor cell-like
R19	astrocytoma	II	mut	Wt	no growth

Supplementary table 5: Patients characteristics (also published in: Sørensen et al., J Neurooncology, 2018).

	N (% of total)
**Age at diagnosis (years)**	61.9 (±13)
**Average survival (months)**	19.3 (±16)
**Overall survival (2 years)**	26%
**Overall survival (5 years)**	3.7%
**Gender**	
- **Male**	123 (57.2%)
- **Female**	92 (42.8%)
**IDH1 R132H status**	
- **Wildtype**	195 (90.7%)
- **Mutated**	20 (9.3%)
**Histological diagnosis (WHO)**	
- **Low-grade glioma (II)**	16 (7.4%)
- **Anaplastic glioma (III)**	15 (7.0%)
- **Glioblastoma (IV)**	184 (85.6%)
**MGMT methylation**	
- **yes**	106 (49.4%)
- **no**	77 (35.8%)
**unknown**	32 (14.8%)
**Seizures of all kind at diagnosis**	
- **yes**	59 (27.4%)
- **no**	151 (70.2%)
- **Uncertain**	5 (2.3%)
**Diagnosis of epilepsy**	
- **yes**	104 (49.3%)
- **no**	106 (48.4%)
- **Uncertain**	6 (2.7%)
- **No sufficient data**	9 (n.a.)

Supplementary Figure 1

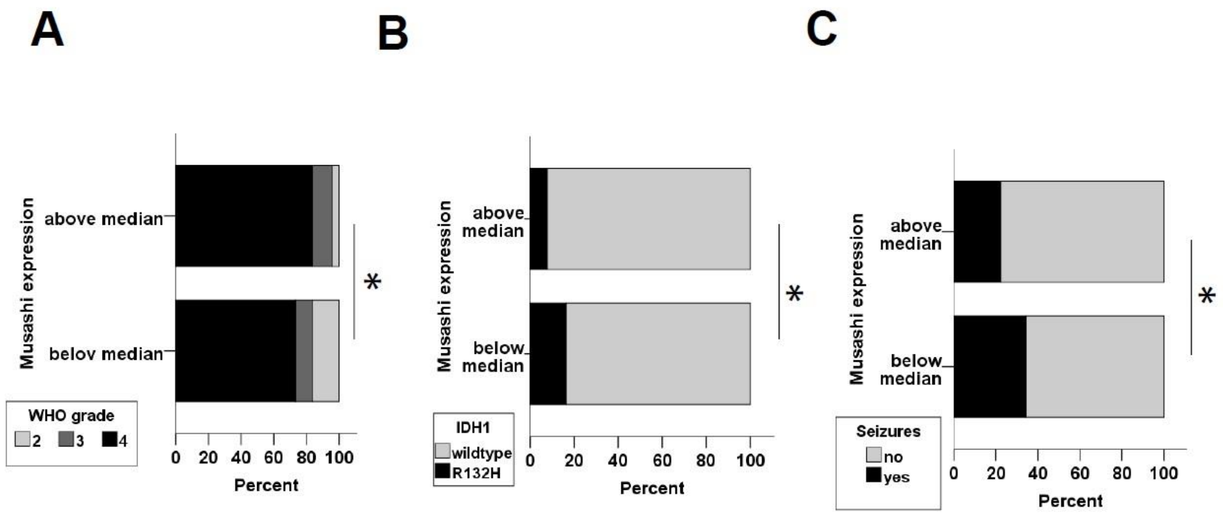

Supplementary Figure 1: Musashi-1 expression and WHO grade, seizures and IDH1 status.
(**A, B**) Distribution of WHO grade (**A**) and IDH mutation status (**B**) in glioma with high and low musashi-1 expression (above and below median, *$p<0.05$, chi^2-test). (**C**) Seizures at onset in tumours with musashi-1 expression above and below median (*$p<0.05$, chi^2-test).

Supplementary Figure 2

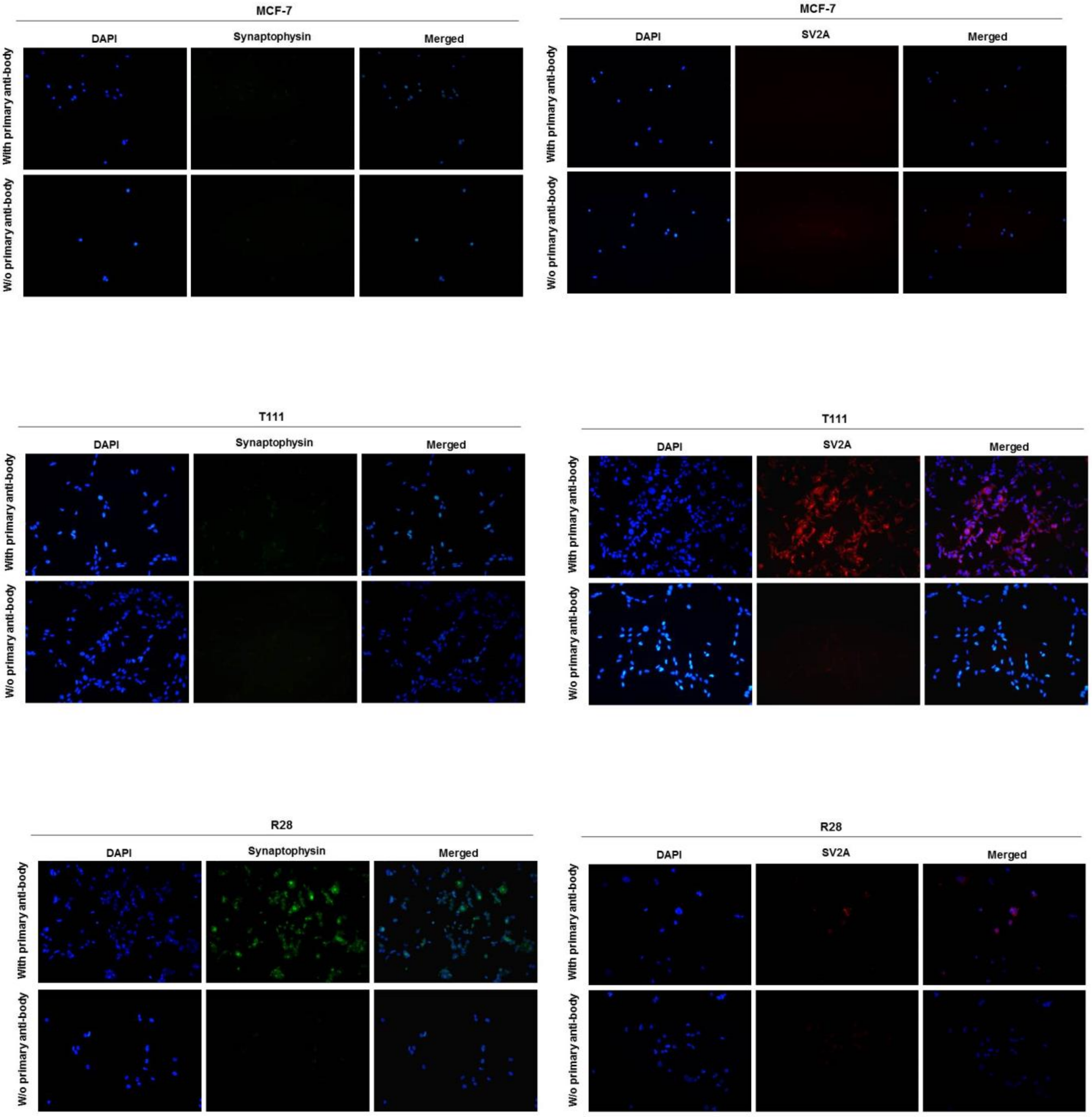

Supplementary Figure 2: Quality control of synaptophysin and SV2A immunostaining.
Breast cancer cells (MCF-7) were used as negative control (upper row). The GSC line T111 was strongly positive for SV2A but not for synaptophysin (2nd row). The GSC line R28 was strongly positive for synaptophysine but not for SV2A (lower row) indicating the specificity of the anti-bodies used.
